# An Unusual Presentation of T-Cell Lymphoblastic Lymphoma with Isolated Renal Involvement

**DOI:** 10.1155/2019/2802141

**Published:** 2019-12-10

**Authors:** Sultan Aydın Köker, Alper Koker, Adem Yasin Köksoy, Yasemin Kayadibi, Ülkü Gül Şiraz, Emine Tekin

**Affiliations:** ^1^Department of Pediatric Hematology and Oncology, SBÜ Van Education and Research Hospital, Van, Turkey; ^2^Department of Pediatric Intensive Care, Hatay State Hospital, Antakya, Hatay, Turkey; ^3^Department of Pediatrics, SBÜ Van Education and Research Hospital, Van, Turkey; ^4^Department of Radiology, SBÜ Van Education and Research Hospital, Van, Turkey

## Abstract

The clinical presentation of Non-Hodgkin lymphoma (NHL) is frequently associated with the involvement of the abdomen and mediastinal lymphadenopathies, but rarely the kidney, ovaries, and testicles. Here, we report a rare case of T-cell lymphoblastic lymphoma (T-LBL) presenting with bilateral nephromegaly without acute renal failure (ARF) as the first manifestation. A 30-month-old boy was admitted to the department of pediatric nephrology exhibiting abdominal distension. Physical examination revealed bilateral renal palpation up to the inguinal region. Elevated lactate dehydrogenase (LDH) levels were detected in his blood. Bilateral diffuse enlarged kidneys with increased hypoechogenicity were found on abdominal ultrasonography. In the next step, contrast-enhanced computed tomography showed diffusely enlarged kidneys, which were compressing the intestinal bowels and midline structures. Renal biopsy demonstrated precursor T-LBL. We wish to report our patient with renal T-LBL presenting with diffuse renal enlargement, which has rarely been reported in the literature.

## 1. Introduction

Non-Hodgkin lymphoma (NHL) is the fifth most common childhood malignancy in children under the age of 15 years, and it accounts for nearly 7% of childhood cancers in developed countries [1]. The median age at diagnosis is approximately ten years, and the incidence increases with age [1]. T-cell lymphoblastic lymphoma (T-LBL) comprises approximately 20% of NHL cases in childhood [2]. Clinically, 75% of patients with T-LBL present with an anterior mediastinal mass, and it may present with lymph node involvement [3]. Primary renal lymphoma (PRL) is described as an NHL involving the kidneys. PRL is very rare (1%) [4]. In the literature, about 70 cases of PRL have been defined, and the large B-cell type comprises the majority of cases [5].

Here, we report a rare case of precursor T-LBL presenting with bilaterally enlarged kidneys and isolated bilateral renal involvement without tumor lysis syndrome or acute renal failure (ARF).

## 2. Case Report

**Figure 1:**
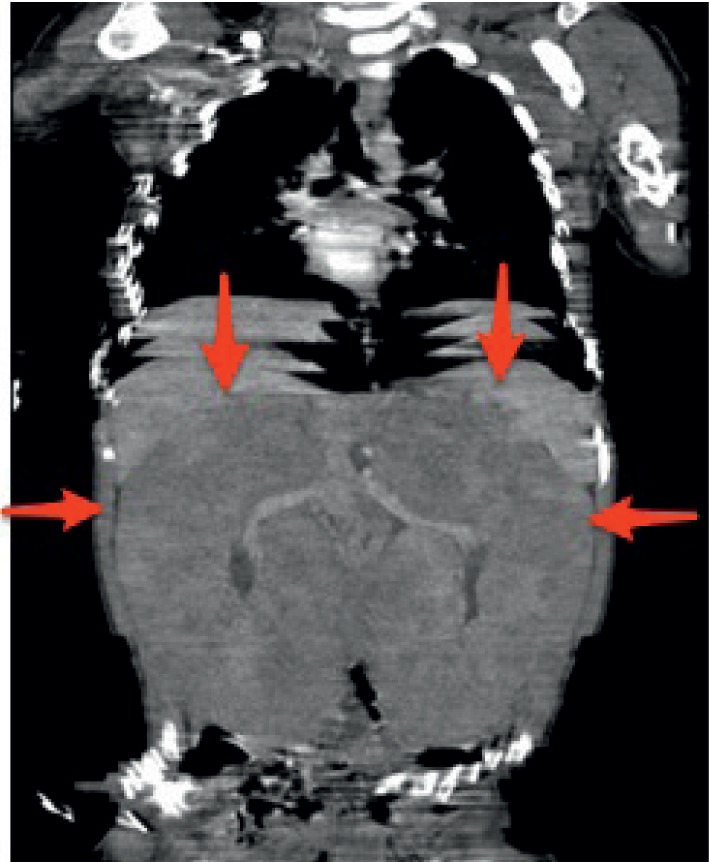
Coronal CT image shows bilaterally enlarged kidneys with decreased enhancement. Enhanced patchy parts are normal renal parenchyma; note the small size of normal parenchyma. Typical for diffuse lymphoma involvement, bilateral kidneys retain their normal bean-like shape.

**Figure 2:**
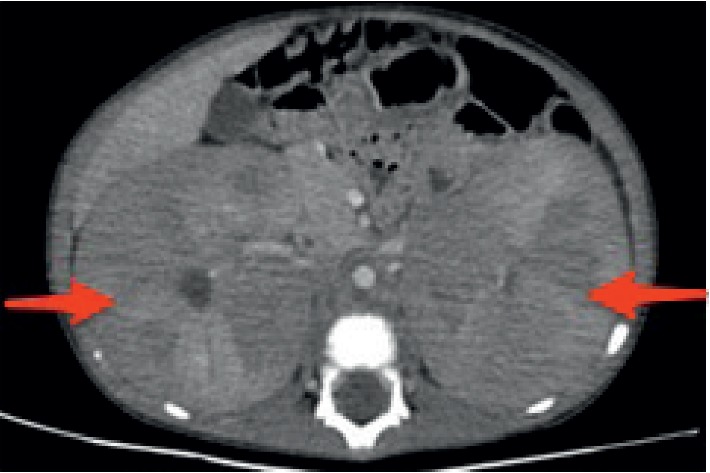
Axial CT image shows diffusely enlarged kidneys. Intestinal bowels and liver are compressed anteriorly.

A 30-month-old, previously healthy male was admitted to the pediatric nephrology department presenting with abdominal distension. Upon physical examination, bilateral renal palpation up to the inguinal region was identified. The chest X-ray did not show a significantly enlarged mediastinum. Abdominal ultrasonography revealed diffuse bilateral renal enlargement. The echogenicity of the kidneys was also decreased and heterogeneous, while the normal bean-shape of the kidneys was preserved and there was no discrete mass formation. Hematological analysis revealed that the patient had a hemoglobin level of 14.7 g/dL, a white blood cell count of 8780 /mm^3^, and a platelet count of 3.6 × 10^9^/L. Serum levels of lactate dehydrogenase (LDH) and uric acid were increased to 1376 U/L and 6 mg/dL, respectively. Additional laboratory findings were normal. Contrast-enhanced computed tomography (CT) scanning showed normally shaped, but diffusely infiltrated kidneys with no normal renal parenchyma. The midline structures, liver, and spleen were also compressed (Figures 1 and 2). Bone marrow biopsy did not detect bone marrow involvement. Our patient was referred to another center for renal biopsy. A diagnostic renal biopsy was performed. The pathological results showed the presence of T-cell lymphoblastic lymphoma, with CD4−/CD8+ phenotype. Immunohistochemistry staining was positive for CD3, CD5, CD7, CD8, and CD1a and negative for CD20, CD79a, CD4, CD10, TdT, and CD99.

On the pediatric tumor committee, NHL-BFM 95 protocol is decided for the chemotherapy regimen [6]. Laboratory findings at the beginning of the treatment were potassium: 4.8 mEq/L, calcium: 9.2 mg/dL, and phosphorus: 3.3 mg/dL. Hyperuricemia, hyperkalemia, hyperphosphatemia, and hypocalcemia occurred on the fifth day of treatment. He had seizures three times, and arrhythmia appeared. After the hydration, urinary alkalinization, and allopurinol treatment started, rasburicase was added. Arrhythmia persisted. Because oliguria and renal failure developed, renal replacement therapy was begun. He died on the eighth day of chemotherapy due to sudden cardiac arrest and acute tumor lysis syndrome.

## 3. Discussion

Lymphomas, which account for approximately 60–70% of adult NHL, form less than 5% of the total childhood NHL. While approximately 55% of all cases with pediatric NHL have B-cell phenotype, 15–20% of them have T-LBL. The majority of children with T-LBL (50–70%) present with rapidly enlarging neck and mediastinal lymphadenopathy. Symptoms often include cough, wheezing, shortness of breath, and orthopnea, though swelling of the neck and face and superior vena cava syndrome can occur due to mediastinal involvement. However, patients can also present with extranodal involvement, including bone, testicles, skin, pancreas, and kidneys [2, 3]. The occurrence of primary renal lymphoma (PRL) is infrequent [7]. Most cases of PRL are of the B-cell type, and only a few cases were reported with the T-cell lineage [5]. Our patient had bilaterally enlarged kidneys. The most interesting aspect of our case is that primary renal involvement was the first presenting symptom of T-LBL. T-LBL occurs more commonly in males. Our patient was a male, in keeping with the literature. The incidence is stable across all pediatric age groups with a median age at diagnosis of 12 years [3]. Thus, our patient was very young according to the literature.

LDH can alter pyruvate to lactate. An increased LDH level suggests more severe tumor progression, tumor angiogenesis, and tumor burden in malignancy [8]. Serum LDH levels may be used in daily clinical practice. Elevated LDH may be a good indicator of cancer in cases like ours with diffuse renal infiltration and bilaterally enlarged kidneys.

For patients with suspected findings, CT is the most sensitive radiological modality, and it can be used for diagnosis, detection of extrarenal extension, staging, and follow-up. Renal lymphoid involvement can be seen as single or multiple masses, an extension of retroperitoneal disease, perirenal disease, or diffuse renal involvement. Preservation of renal shape in the infiltrative form could create challenges for the diagnosis [9]. Our patient had diffuse renal involvement.

The exploration of extranodal sites or excisional node biopsy is commonly performed for diagnosis. Immunohistochemical studies are also required to diagnose T-LBL. T-LBL is confirmed when lineage markers for B-cells (CD20) and NK cells (CD56) are negative and those for precursor T-cells (CD3 and CD5) are positive [10]. T-LBL was detected as a result of biopsy in our case because TdT and T-cell markers (CD3) were positive based on immunohistochemical analysis of tumor cells.

In a study of the German BFM group, the 5-year event-free survival was estimated at 90% among children who were given an ALL-type chemotherapy regimen [11]. Moreover, systemic chemotherapy can use a CHOP regimen for treatment. Median survival is usually <1 year [12], but the addition of rituximab to the combination chemotherapy has improved the survival rate [13]. Because patients with T-LBL have poorer outcomes despite this chemotherapy regimen and it is hard to fight tumor lysis syndrome and renal failure at the same time, our patient died on the eighth day of the NHL-BFM 95 protocol.

The clinical presentation of our patient with bilaterally enlarged kidneys, diffuse renal infiltration, and elevated LDH suggested a diagnosis of isolated renal involvement of lymphoma. Histopathological examination of the renal biopsy with immunohistochemical study determined the diagnosis of T-LBL. Biopsy and histopathology are essential for a definite diagnosis of lymphoma. We wished to report our case of T-LBL presenting with rare renal involvement.
